# Supplementary material for: RNA polymerase II pausing factor NELF in CD8+ T cells promotes antitumor immunity
Source: Nat Commun. 2022 Apr 20;13:2155. doi: 10.1038/s41467-022-29869-2 (PMC9021285; doi:10.1038/s41467-022-29869-2)
Supplement: Supplementary file 1 — supplementary information [file 41467_2022_29869_MOESM1_ESM.pdf]

Supplementary Information

RNA Polymerase II Pausing Factor NELF in $CD8^+$ T Cells Promotes Antitumor Immunity

Bogang Wu[1#], Xiaowen Zhang[1#], Huai-Chin Chiang[1], Haihui Pan[1], Bin Yuan[1], Payal Mitra[2], Leilei Qi[2], Hayk Simonyan[3], Colin N. Young[3], Eric Yvon[4], Yanfen Hu[2], Nu Zhang[5], Rong Li[1*]

[1]Department of Biochemistry & Molecular Medicine,
[2]Department of Anatomy & Cell Biology,
[3]Department of Pharmacology & Physiology,
[4]Department of Medicine
The George Washington University Cancer Center
School of Medicine & Health Sciences,
The George Washington University, Washington, DC 20037, USA
[5]Department of Microbiology, Immunology & Molecular Genetics,
University of Texas Health San Antonio, San Antonio, TX 78229, USA

[#]These authors contributed equally to the work
[*]Correspondence to: R.L. (rli69@gwu.edu)

Supplementary Figure 1

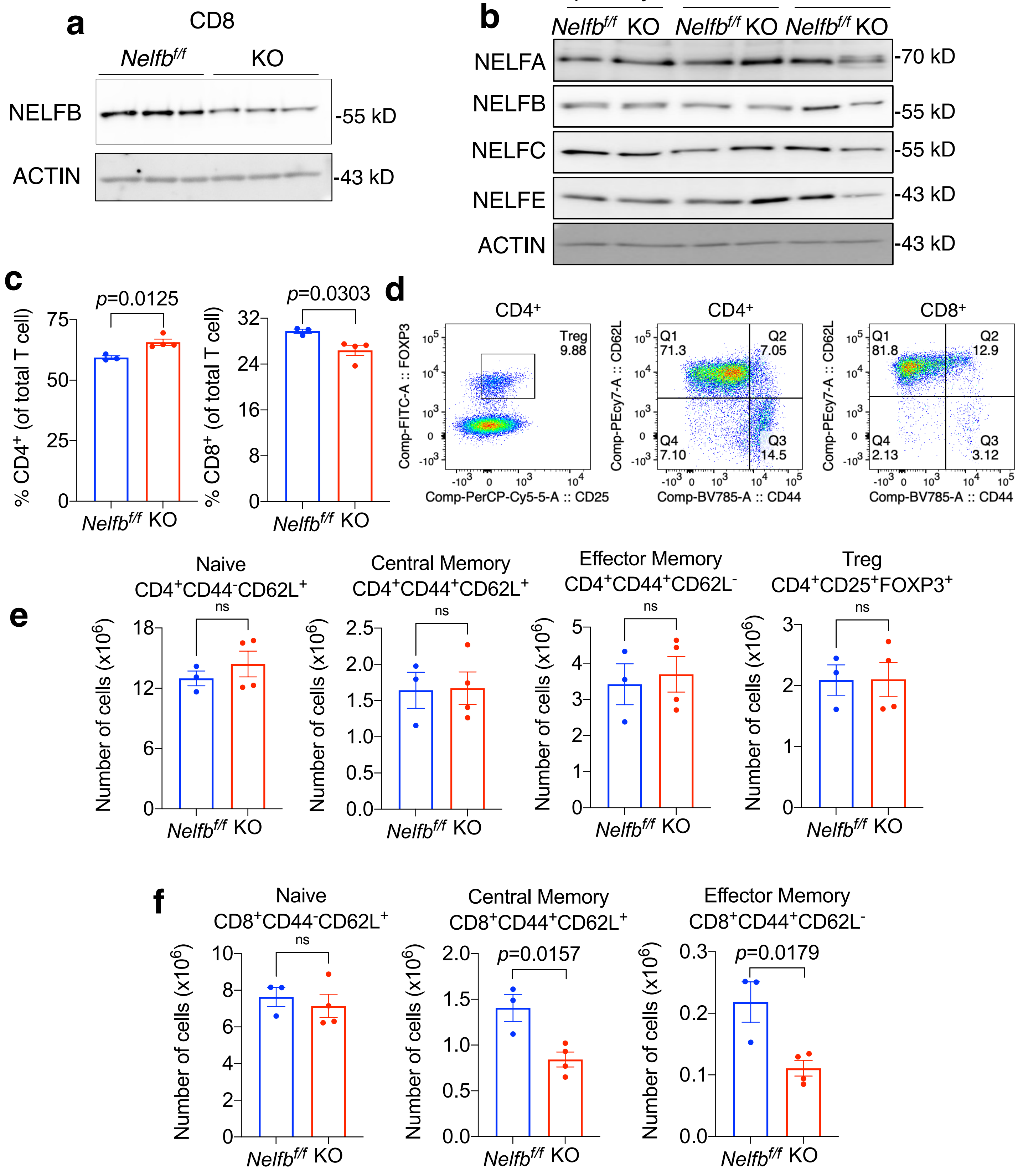

Supplementary Figure 1. *Nelfb* deletion impairs memory CD8$^+$ T cell in tumor-free mice. (**a**) Western blots for NELFB and ACTIN in CD8$^+$ T cells isolated from *Nelfb$^{f/f}$* and KO mice splenocytes. (**b**) Western blot for NELFA, NELFB, NELFC, NELFE, and ACTIN in total splenocytes, purified CD8$^+$ T cells, or non-CD8$^+$ cells from splenocytes of *Nelfb$^{f/f}$* and KO mice. The experiments were independently repeated three times with similar results (a, b). (**c**) Flow cytometry analysis of total CD4$^+$ (% of total T cells) and CD8$^+$ (% of total T cells); *Nelfb$^{f/f}$* (n=3), KO (n=4). (**d**) Representative flow gating strategy for mouse splenocytes. (**e**) Cell counts for different subsets of CD4$^+$ T cells from mouse spleen; *Nelfb$^{f/f}$* (n=3), KO (n=4). (**f**) Cell counts for different subsets of CD8$^+$ T cells from mouse spleen; *Nelfb$^{f/f}$* (n=3), KO (n=4). Baseline flow-based immunophenotyping were done using mice at 16-week age. All data are presented as mean values +/- SEM; two-sided Student's t-test. Source data are provided as a Source Data file.

Supplementary Figure 2

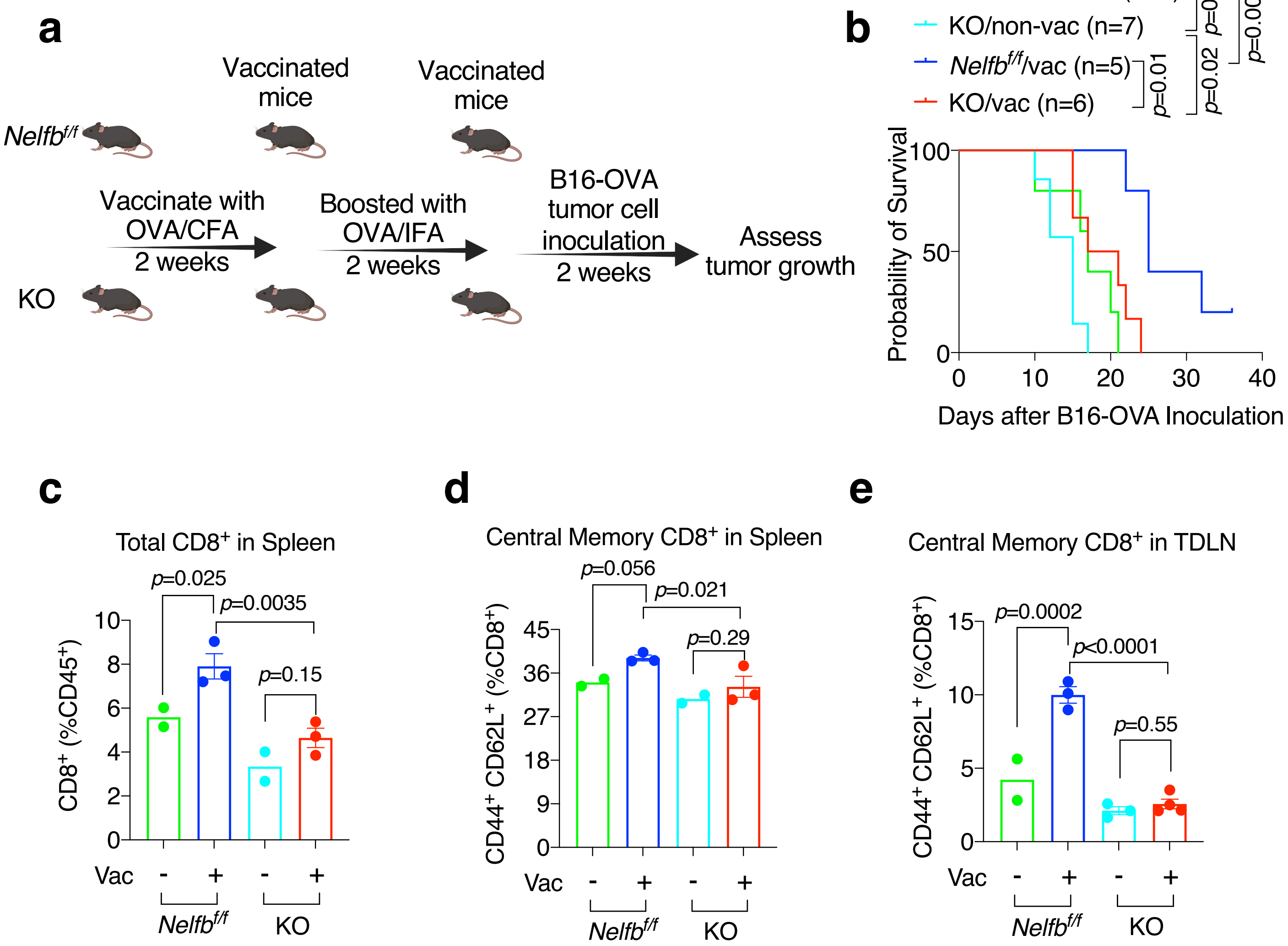

Supplementary Figure 2. *Nelfb* deletion in T cells impairs memory response to tumor antigen vaccination. (**a**) Scheme of vaccination procedure using OVA protein followed by B16-OVA tumor challenge. (**b**) Survival curve of B16-OVA tumor-bearing *Nelfb$^{f/f}$* and KO mice with or without vaccination; Gehan-Breslow-Wilcoxon test. (**c-e**) Immunophenotyping of B16 tumor-bearing mice by flow cytometry analysis; for spleen: *Nelfb$^{f/f}$* non-vac (n=2), *Nelfb$^{f/f}$* vac (n=3), KO non-vac (n=2), KO vac (n=3); for tumor draining lymph nodes (TDLN): *Nelfb$^{f/f}$* non-vac (n=2), *Nelfb$^{f/f}$* vac (n=3), KO non-vac (n=3), KO vac (n=4). (c) $CD8^+$ (% of $CD45^+$) cells in spleen; (d) central memory $CD44^+CD62L^+$ (% of $CD8^+$) cells in spleen; (e) central memory $CD44^+CD62L^+$ (% of $CD8^+$) cells in TDLN. Data were presented as mean +/- SEM; one-way-ANOVA. Source data are provided as a Source Data file.

Supplementary Figure 3

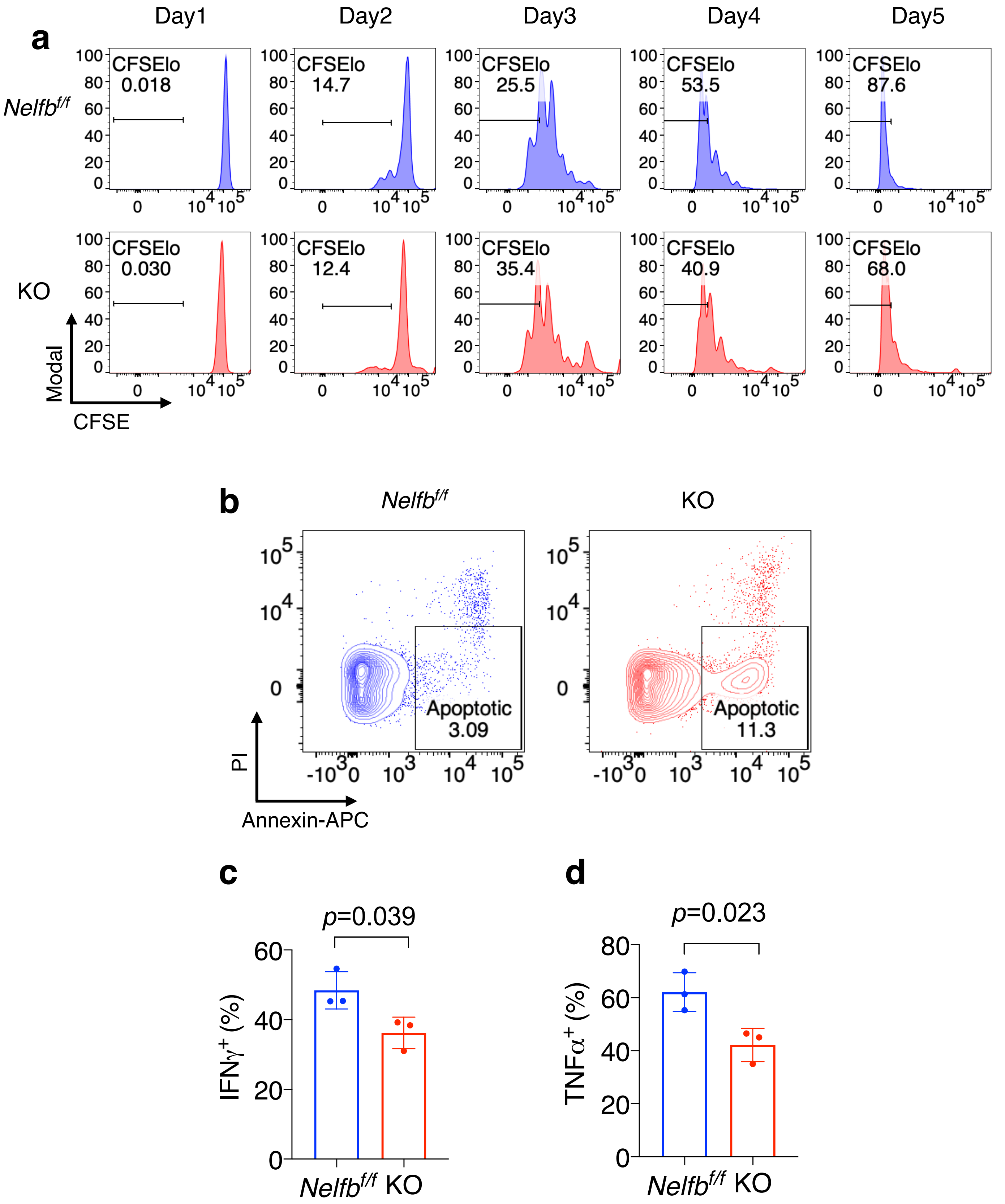

Supplementary Figure 3. *Nelfb* deletion effects on apoptosis and proliferation of CD8+ T cells. (a) Representative flow cytometry plot of CFSE labeling assay for *Nelfb*$^{f/f}$ and KO CD8+ T cells during *in vitro* proliferation. (**b**) Representative flow cytometry plot of annexin/PI staining for WT/KO CD8+ T cells following anti-CD3/CD28 activation. (**c-d**) Quantification of IFNγ+ (c) and TNFα+ (d) cell percentages after *ex vivo* expansion, n=3/group. All data are presented as mean values +/- SD; two-sided Student's t-test. Source data are provided as a Source Data file.

Supplementary Figure 4

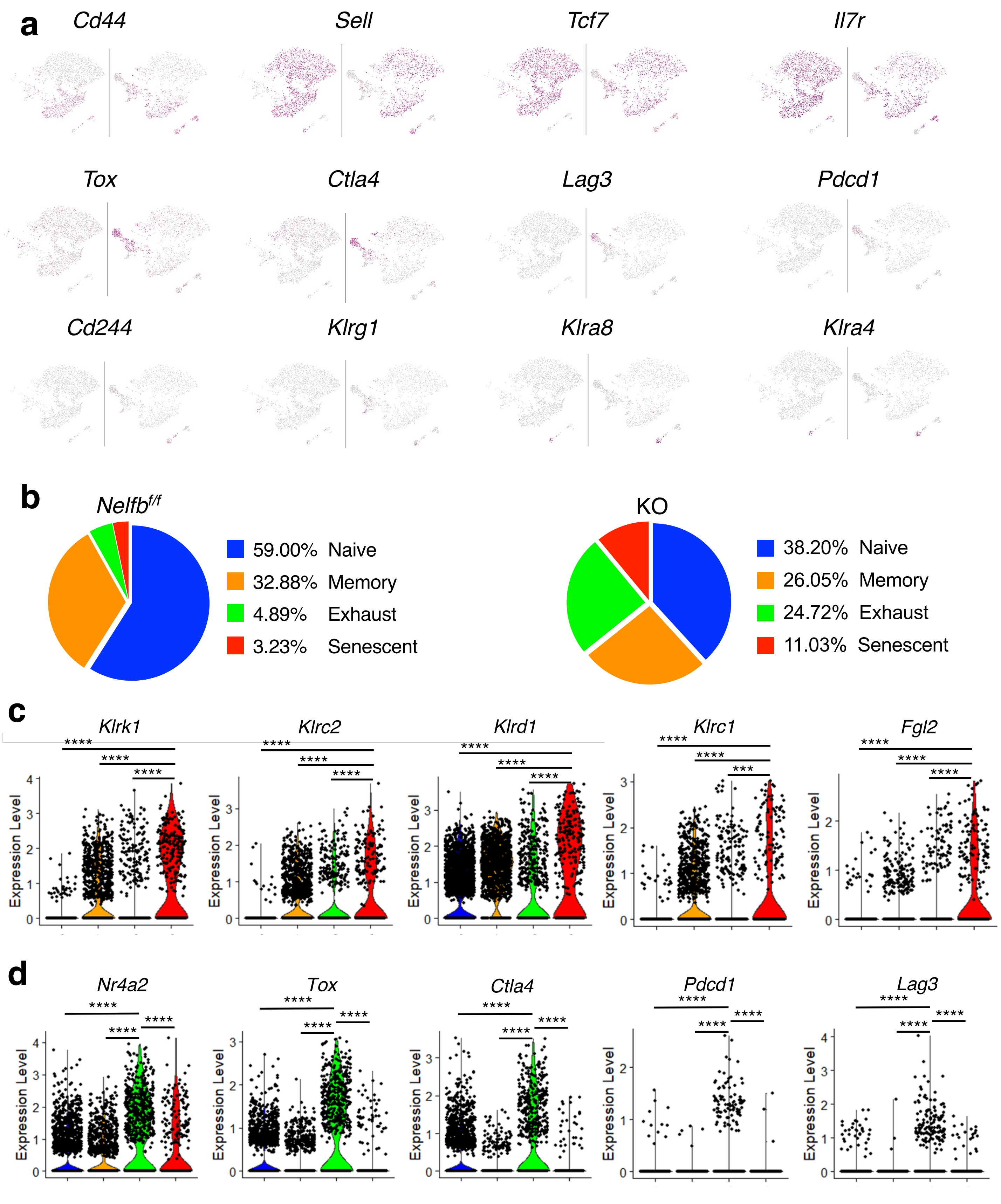

Supplementary Figure 4. Marker gene expression used in defining distinct populations for scRNA-seq. (**a**) Selective marker gene expression profiles in different clusters of scRNA-seq; left: *Nelfb*$^{f/f}$, right: KO. (**b**) Population distribution for *Nelfb*$^{f/f}$ and KO CD8^{+} cells from scRNA-seq. (**c-d**) Violin plots for senescence (c) and exhaustion (d) marker gene expression among subset populations. **** denotes $p<0.0001$; *** denotes $0.001<p<0.0001$; Likelihood-ratio test for single cell feature expression.

Supplementary Figure 5

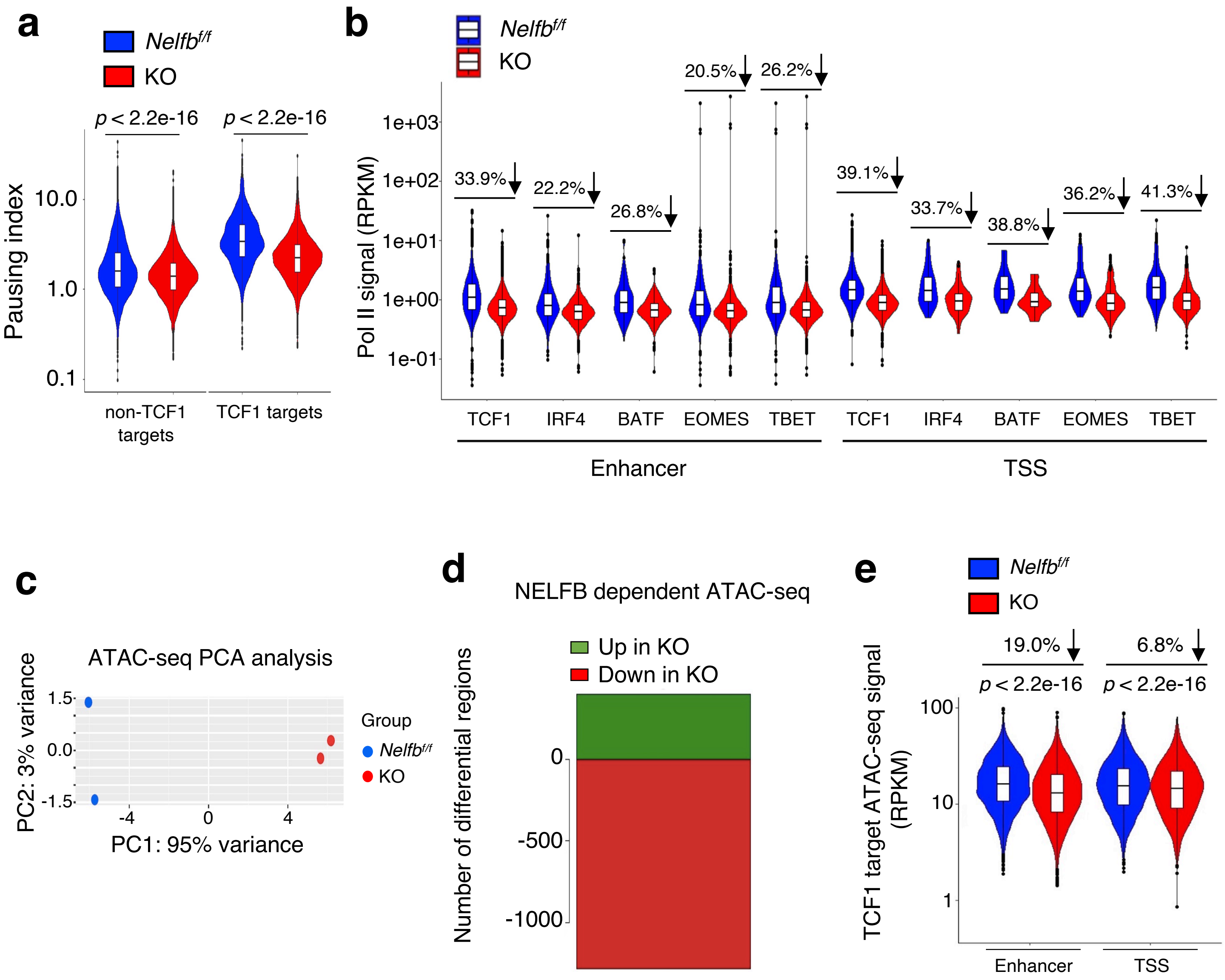

Supplementary Figure 5. Differences in NELF-dependent Pol II ChIP-seq and ATAC-seq. (**a**) Pol II pausing index from *Nelfb*$^{f/f}$ and KO CD8^{+} T cells; non-TCF targets (n=27654), TCF1 targets (n=4297). (**b**) RNA Pol II signal by TFs (using published ChIP-seq data in T cells) and locations. (**c**) Principal component analysis of ATAC-seq datasets for *Nelfb*$^{f/f}$ and KO CD8^{+} T cells. (**d**) Differential regions on ATAC-seq for *Nelfb*$^{f/f}$ and KO CD8^{+} T cells. (**e**) ATAC-seq signals at enhancer and TSS regions of TCF1 targets of *Nelfb*$^{f/f}$ and KO CD8^{+} T cells; enhancer (n=3901), TSS (n=2768). The bounds of the box represent the 25th and 75th percentiles of the interquartile range. The black line in the box interior represents the median of the data. The whiskers represent the minimum and maximum values of the data and the black dot outside the box and whiskers represents an outlier. Violin plots were assessed by two-sided Wilcoxon rank sum test with continuity correction. Source data are provided as a Source Data file.

Supplementary Figure 6

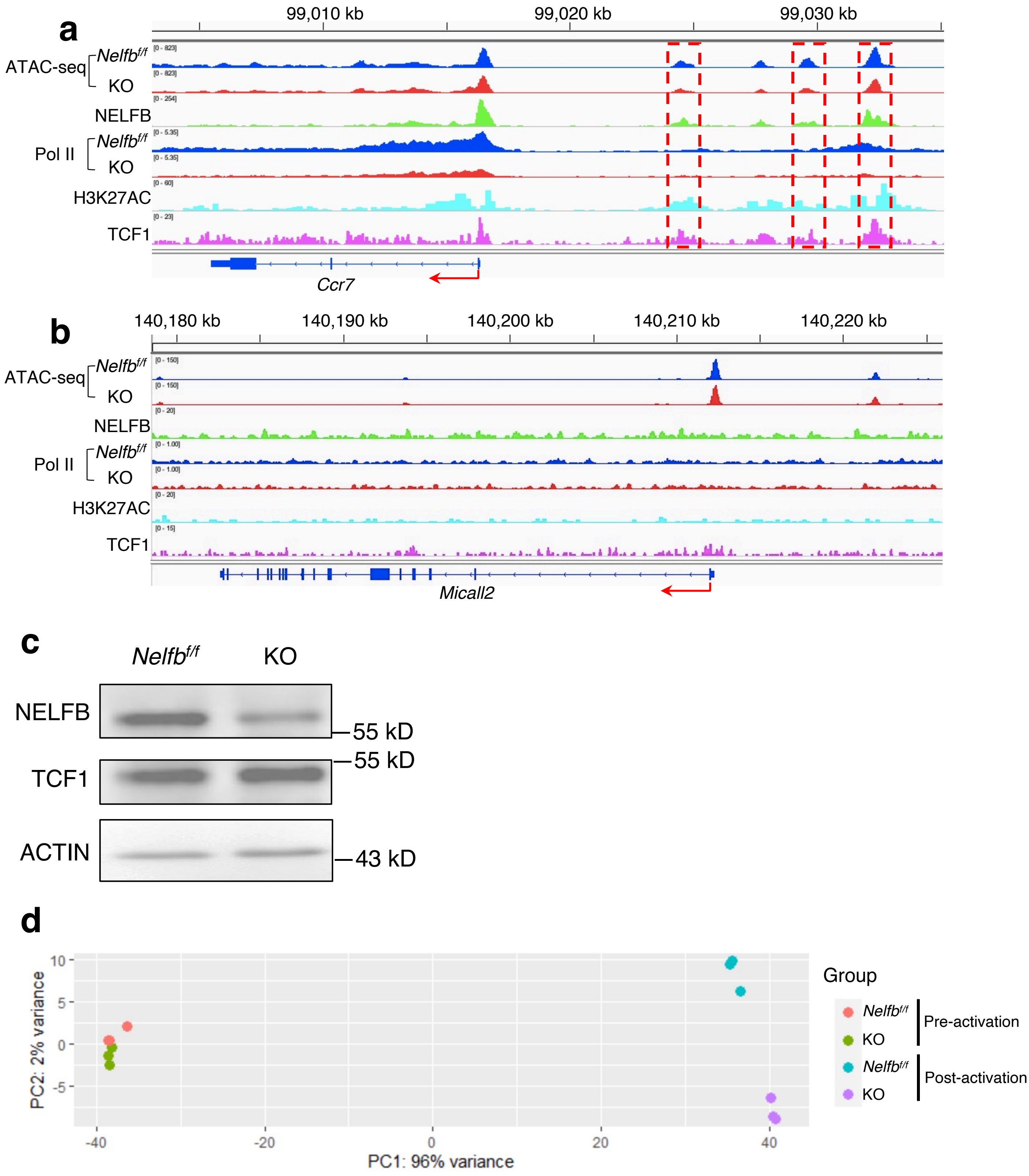

Supplementary Figure 6. ATAC-seq and transcriptomic differences of *Nelfb*$^{f/f}$ and KO CD8^{+} cells. (a-b) IGV views of ChIP-seq and ATAC-seq tracks for *Ccr7*, a key memory related gene (a); and *Micall2*, a non-T cell-related gene, was shown as a negative control (b). (**c**) Representative Western blot for NELFB, TCF1 and ACTIN in *Nelfb*$^{f/f}$ and KO CD8^{+} T cells. The experiments were independently repeated three times with similar results. (**d**) Principal component analysis for bulk RNA-seq. Source data are provided as a Source Data file.

Supplementary Figure 7

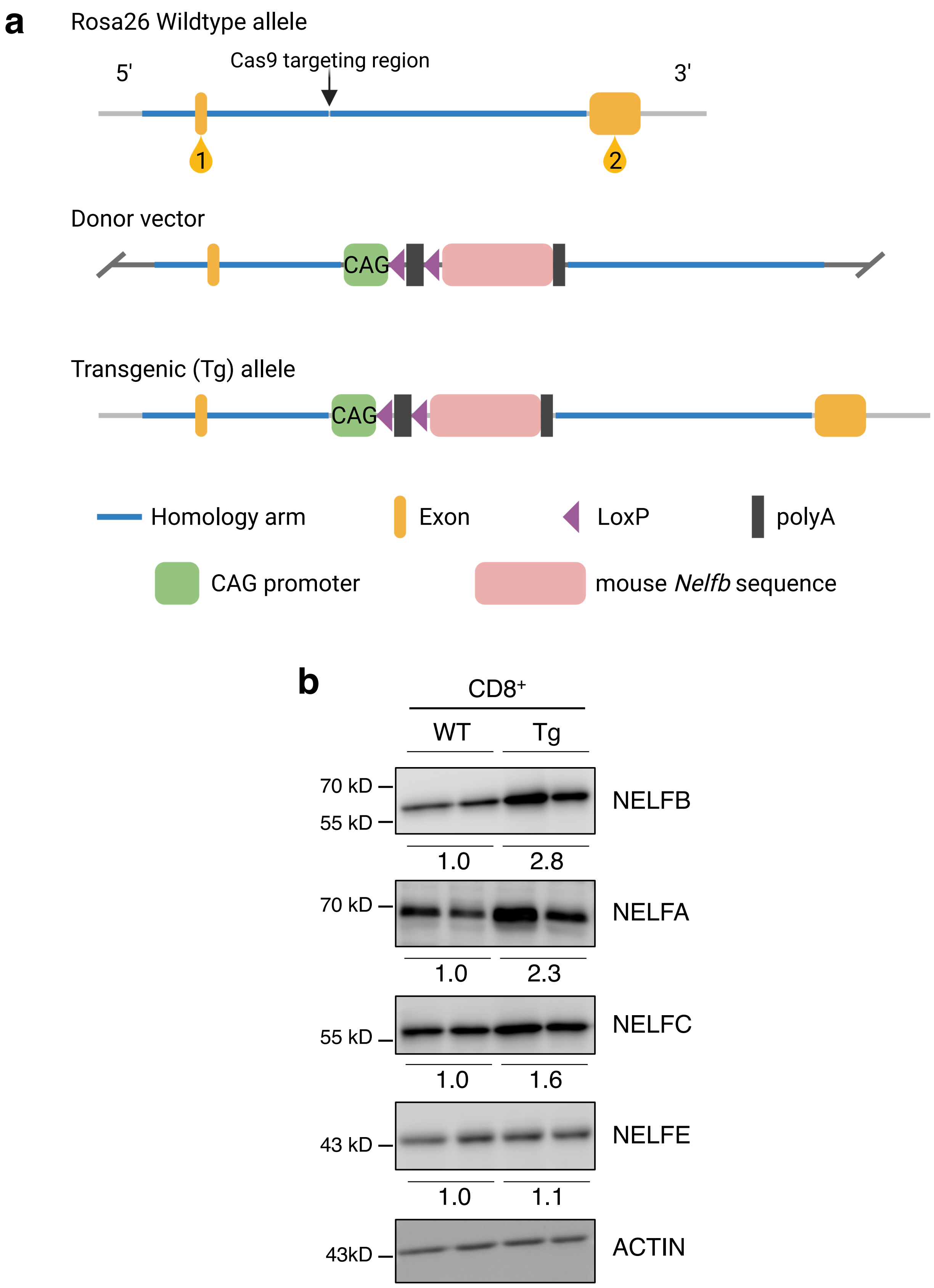

Supplementary Figure 7. *Nelfb* transgenic mouse model. (**a**) Strategy used for generating *Nelfb* transgenic mouse model (see methods for details). (**b**) Expression of other NELF subunits in *Nelfb* transgenic mouse spleen CD8$^+$ T cells. The experiments were independently repeated twice with similar results. Source data are provided as a Source Data file.

Supplementary Figure 8

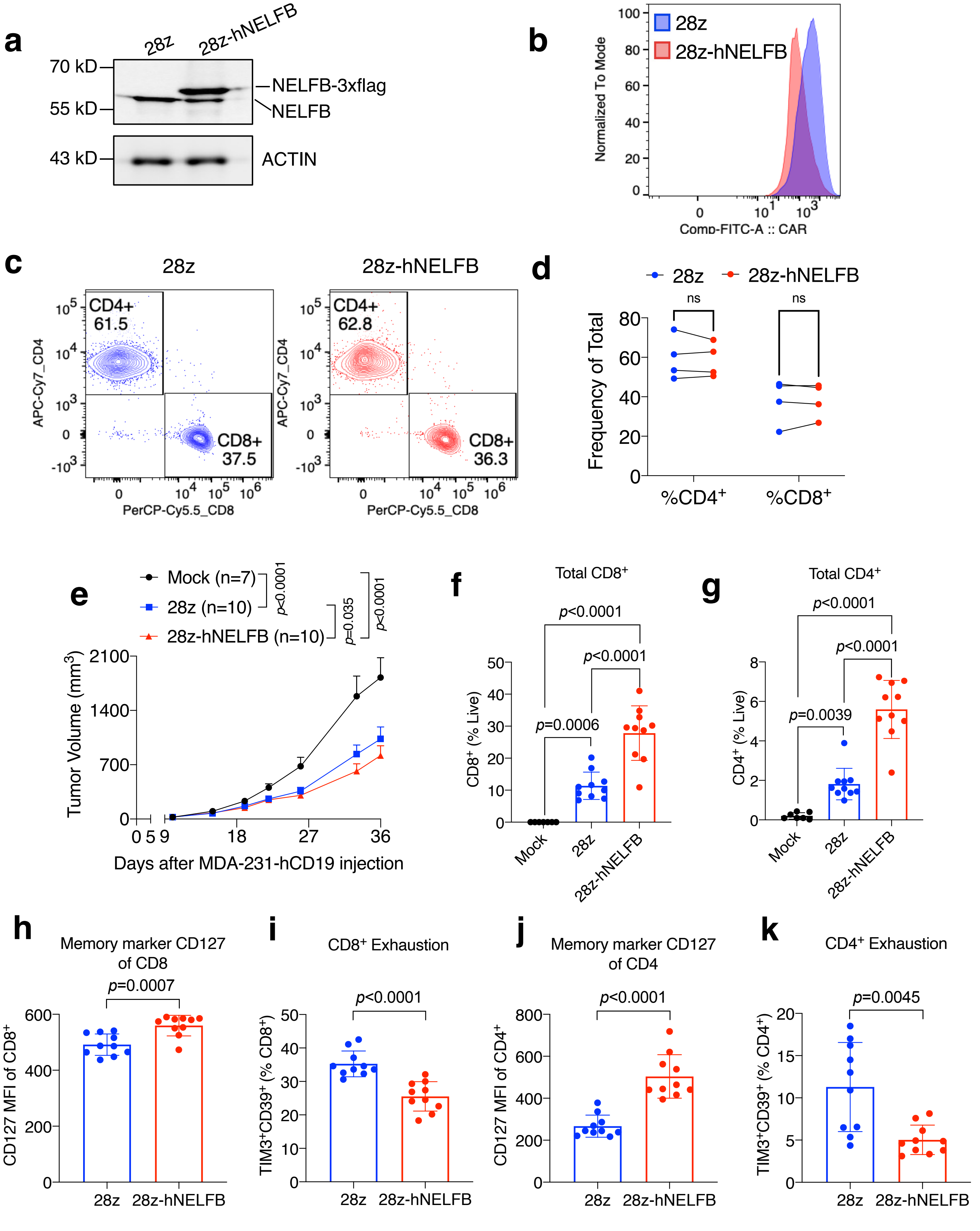

Supplementary Figure 8. NELFB overexpression boosts human CAR-T efficacy. (**a**) Representative Western blots for NELFB and ACTIN in CAR-T cells with or without *NELFB* overexpression. The experiments were independently repeated twice with similar results. (**b**) CAR detection by FITC-labeled human CD19. (**c**) Representative flow cytometry plot of CD4 and CD8 staining for CAR-T cells after *in vitro* expansion. (**d**) Frequency of total CAR-T cell for $CD4^+$ and $CD8^+$ T cells after *in vitro* expansion, n=4/group. (**e**) Tumor growth curves for MDA-231-hCD19; Mock (n=7), 28z (n=10), 28z-hNELFB (n=10); data are presented as mean values +/- SEM. (**f-k**) MDA-231-hCD19 TILs analysis for percentages of $CD8^+$ (f), percentages of $CD4^+$ (g), CD127 MFI of $CD8^+$ (h), percentages of exhausted $CD8^+$ (i), CD127 MFI of $CD4^+$ (j), and percentage of exhausted $CD4^+$ (k); Mock (n=7), 28z (n=10), 28z-hNELFB (n=10); data are presented as mean values +/- SD; Student's t-test (for two groups) or one-way-ANOVA (for three or more groups). Tumor curves were compared using two-way-ANOVA followed by multiple comparisons. Two-sided tests were used. Source Data are provided as a Source Data file.

Supplementary Figure 9

a

Sequencing QC	*Nelfb*$^{f/f}$	KO
Number of Reads	241,202,687	167,732,290
Valid Barcodes	97.0%	96.8%
Valid UMIs	99.9%	100.0%
Sequencing Saturation	80.0%	79.2%
Q30 Bases in Barcode	96.8%	96.8%
Q30 Bases in RNA Read	90.2%	90.0%
Q30 Bases in Sample Index	94.7%	94.4%
Q30 Bases in UMI	96.9%	96.9%

b

Mapping QC	*Nelfb*$^{f/f}$	KO
Reads Mapped to Genome	94.6%	94.3%
Reads Mapped Confidently to Genome	91.5%	91.4%
Reads Mapped Confidently to Intergenic Regions	5.9%	6.1%
Reads Mapped Confidently to Intronic Regions	25.1%	26.8%
Reads Mapped Confidently to Exonic Regions	60.5%	58.5%
Reads Mapped Confidently to Transcriptome	56.0%	54.0%
Reads Mapped Antisense to Gene	2.0%	2.0%

c

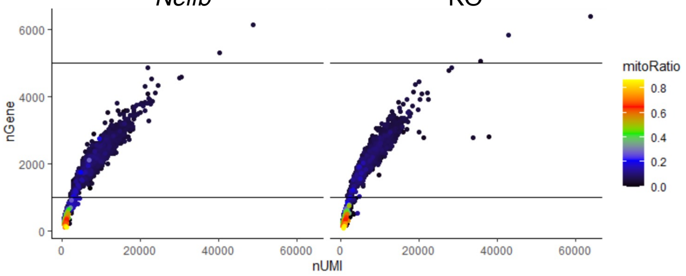

d

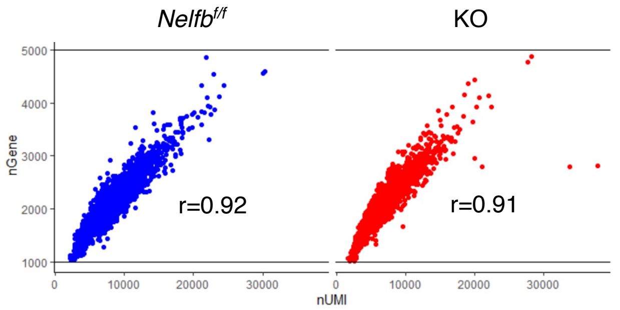

e

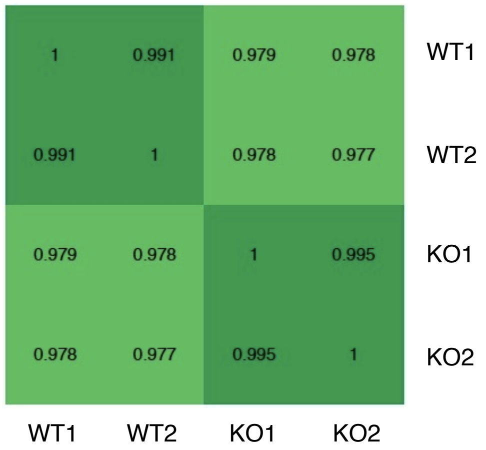

Supplementary Figure 9. QC for systems data. (**a-b**) Sequencing (a) and Mapping (b) QC for scRNA-seq. (**c**) QC metrics of number of UMI (x axis), number of gene (y axis) and mitochondrial gene percentage (color). Black lines mark the filter criteria. (**d**) Post filter QC showing the correlation between number of UMI and number of genes detected. (**e**) Pearson correlation statistics are shown for peak read numbers correlation heatmap for ATAC-seq.
