# Supplementary material for: RNA polymerase II pausing factor NELF in CD8+ T cells promotes antitumor immunity
Source: Nat Commun. 2022 Apr 20;13:2155. doi: 10.1038/s41467-022-29869-2 (PMC9021285; doi:10.1038/s41467-022-29869-2)
Supplement: Supplementary file 2 — Reporting Summary [file 41467_2022_29869_MOESM2_ESM.pdf]

nature portfolio

Corresponding author(s): Rong Li

Last updated by author(s): Mar 23, 2022

Reporting Summary

Nature Portfolio wishes to improve the reproducibility of the work that we publish. This form provides structure for consistency and transparency in reporting. For further information on Nature Portfolio policies, see our Editorial Policies and the Editorial Policy Checklist.

Statistics

For all statistical analyses, confirm that the following items are present in the figure legend, table legend, main text, or Methods section.

n/a	Confirmed	
☐	☒	The exact sample size (*n*) for each experimental group/condition, given as a discrete number and unit of measurement
☐	☒	A statement on whether measurements were taken from distinct samples or whether the same sample was measured repeatedly
☐	☒	The statistical test(s) used AND whether they are one- or two-sided<br>*Only common tests should be described solely by name; describe more complex techniques in the Methods section.*
☒	☐	A description of all covariates tested
☐	☒	A description of any assumptions or corrections, such as tests of normality and adjustment for multiple comparisons
☐	☒	A full description of the statistical parameters including central tendency (e.g. means) or other basic estimates (e.g. regression coefficient) AND variation (e.g. standard deviation) or associated estimates of uncertainty (e.g. confidence intervals)
☐	☒	For null hypothesis testing, the test statistic (e.g. *F*, *t*, *r*) with confidence intervals, effect sizes, degrees of freedom and *P* value noted<br>*Give P values as exact values whenever suitable.*
☒	☐	For Bayesian analysis, information on the choice of priors and Markov chain Monte Carlo settings
☒	☐	For hierarchical and complex designs, identification of the appropriate level for tests and full reporting of outcomes
☒	☐	Estimates of effect sizes (e.g. Cohen's *d*, Pearson's *r*), indicating how they were calculated

Our web collection on statistics for biologists contains articles on many of the points above.

Software and code

Policy information about availability of computer code

Data collection	Flow cytometry data were collected using BD Celesta analyzer, 3-laser, 12-color.Immunoblotting images were collected using ChemiDoc Touch Imaging System (BIO-RAD)
Data analysis	Flow cytometry data were analyzed using FlowJo (FlowJo, version 10.6) and BD FACSDiva Software (v9.0). Statistical analysis was performed using Graphpad Prism (Graphpad Software, version 8). Gene sets enrichment analysis were performed using GSEA software (GSEA_4.1.0). ChIP-seq data quality was checked by MultiQC (v1.9).

For manuscripts utilizing custom algorithms or software that are central to the research but not yet described in published literature, software must be made available to editors and reviewers. We strongly encourage code deposition in a community repository (e.g. GitHub). See the Nature Portfolio guidelines for submitting code & software for further information.

Data

Policy information about availability of data

All manuscripts must include a data availability statement. This statement should provide the following information, where applicable:

- Accession codes, unique identifiers, or web links for publicly available datasets
- A description of any restrictions on data availability
- For clinical datasets or third party data, please ensure that the statement adheres to our policy

All data are available in the main text or the supplementary materials. The sequencing data that support the findings of this study have been deposited to NCBI Gene Expression Omnibus under accession codes GSE182862 [https://www.ncbi.nlm.nih.gov/geo/query/acc.cgi?acc=GSE182862]. Human melanoma tumor-infiltrating lymphocytes (TIL) data were downloaded from GSE7205650. Source data are provided with this paper.

Field-specific reporting

Please select the one below that is the best fit for your research. If you are not sure, read the appropriate sections before making your selection.

- [x] Life sciences
- [ ] Behavioural & social sciences
- [ ] Ecological, evolutionary & environmental sciences

For a reference copy of the document with all sections, see nature.com/documents/nr-reporting-summary-flat.pdf

Life sciences study design

All studies must disclose on these points even when the disclosure is negative.

Sample size	Sample size is determined by pilot experiments and resource availability.
Data exclusions	No data exclusion.
Replication	The number of biological replicates were reported in the figure legends. Results from representative experiments were confirmed in at least two independent experiment repeats.
Randomization	Mice were randomized and exposed to the same environment. For in vitro cell culture based and biochemical related experiments, samples with different genotypes were treated equally and analyzed at same time.
Blinding	Tumor measurements were confirmed by a second person who is blinded to the genotype or treatment information. For key in vitro cell culture based and biochemical related experiments, analyses were confirmed by second person who is blinded to the genotype information.

Reporting for specific materials, systems and methods

We require information from authors about some types of materials, experimental systems and methods used in many studies. Here, indicate whether each material, system or method listed is relevant to your study. If you are not sure if a list item applies to your research, read the appropriate section before selecting a response.

Materials & experimental systems

n/a	Involved in the study	
☐	☒	Antibodies
☐	☒	Eukaryotic cell lines
☒	☐	Palaeontology and archaeology
☐	☒	Animals and other organisms
☒	☐	Human research participants
☒	☐	Clinical data
☒	☐	Dual use research of concern

Methods

n/a	Involved in the study	
☐	☒	ChIP-seq
☐	☒	Flow cytometry
☒	☐	MRI-based neuroimaging

Antibodies

Antibodies used

Antibodies used in the flow cytometry assays were anti-CD45 (Invitrogen, 11-0451-82), anti-CD3 (Tonbo Biosciences, 65-0031-U100), anti-CD8 (BD Pharmingen, 557654), anti-CD44 (BioLegend, 103041), anti-CD62L (BioLegend, 104424), anti-PD1 (BD Biosciences, 563059), anti-TIM3 (BioLegend, 119704), anti-LAG3 (Invitrogen, 25223182), anti-IFNg (BioLegend, 505826) and anti-TNFa (BioLegend, 506314), anti-CD4 (BioLegend, 317418), anti-CD8 (BioLegend, 344710), anti-CD45RA (BioLegend, 304112), anti-CD62L (BioLegend, 304806), and anti-CD19 antibody (Biolegend, 302239).
Antibodies used in the western blotting assays were anti-TCF1 (CST, 2203S), anti-NELFE (Proteintech, 10705-1-AP), anti-GAPDH (Bio-Rad, 12004167), anti-NELFB (Cell Signaling Technology, 14894S), anti-NELFA (Proteintech, 10456-1-AP), and anti-NELFC (Cell Signaling Technology, 12265S).
Antibodies used in the ChIP-seq assays were anti-Pol II (BioLegend; 664906) and anti-NELFB (Cell Signaling Technology;14894S).

Validation

All antibodies were validated by the manufacturers and used at the dilutions suggested by the manufacturers.
anti-CD45 (Invitrogen, 11-0451-82): https://www.thermofisher.com/antibody/product/CD45-Antibody-clone-30-F11-Monoclonal/11-0451-82,
anti-CD3 (Tonbo Biosciences, 65-0031-U100): https://tonbobio.com/products/percp-cyanine5-5-anti-mouse-cd3e-145-2c11,
anti-CD8 (BD Pharmingen, 557654): https://www.bdbiosciences.com/en-us/products/reagents/flow-cytometry-reagents/research-reagents/single-color-antibodies-ruo/apc-cy-7-rat-anti-mouse-cd8a.557654,
anti-CD44 (BioLegend, 103041): https://www.biolegend.com/en-us/search-results/brilliant-violet-785-anti-mouse-human-cd44-antibody-7959,
anti-CD62L (BioLegend, 104424): https://www.biolegend.com/en-us/search-results/pacific-blue-anti-mouse-cd62l-antibody-3117,
anti-PD1 (BD Biosciences, 563059): https://www.bdbiosciences.com/en-sg/products/reagents/flow-cytometry-reagents/research-reagents/single-color-antibodies-ruo/bv605-hamster-anti-mouse-cd279-pd-1.563059,

anti-TIM3 (BioLegend, 119704): https://www.biolegend.com/en-us/products/pe-anti-mouse-cd366-tim-3-antibody-2657?GroupID=BLG10656,
anti-LAG3 (Invitrogen, 25223182): https://www.thermofisher.com/antibody/product/CD223-LAG-3-Antibody-clone-eBioC9B7W-C9B7W-Monoclonal/25-2231-82,
anti-IFNg (BioLegend, 505826): https://www.biolegend.com/en-us/search-results/pe-cyanine7-anti-mouse-ifn-gamma-antibody-5865,
anti-TNFa (BioLegend, 506314): https://www.biolegend.com/en-us/products/alexa-fluor-647-anti-mouse-tnf-alpha-antibody-2724,
anti-CD4 (BioLegend, 317418): https://www.biolegend.com/en-us/products/apc-cyanine7-anti-human-cd4-antibody-3658,
anti-CD8 (BioLegend, 344710): https://www.biolegend.com/en-us/products/percp-cyanine5-5-anti-human-cd8-antibody-6389,
anti-CD45RA (BioLegend, 304112): https://www.biolegend.com/en-us/products/apc-anti-human-cd45ra-antibody-684,
anti-CD62L (BioLegend, 304806): https://www.biolegend.com/en-us/products/pe-anti-human-cd62l-antibody-653,
and anti-CD19 antibody (Biolegend, 302239): https://www.biolegend.com/en-us/products/brilliant-violet-785-anti-human-cd19-antibody-7967,
anti-TCF1 (CST, 2203S): https://www.cellsignal.com/products/primary-antibodies/tcf1-tcf7-c63d9-rabbit-mab/2203,
anti-NELFE (Proteintech, 10705-1-AP): https://www.ptglab.com/products/RDBP-Antibody-10705-1-AP.htm,
anti-GAPDH (Bio-Rad, 12004167): https://www.bio-rad.com/en-us/sku/12004167-hfab-rhodamine-anti-gapdh-primary-antibody-200-ul?ID=12004167,
anti-Pol II (BioLegend; 664906): https://www.biolegend.com/en-us/search-results/purified-anti-rna-polymerase-ii-rpb1-antibody-11666,
anti-NELFB (Cell Signaling Technology;14894S): https://www.cellsignal.com/products/primary-antibodies/cobra1-d6k9a-rabbit-mab/14894,
anti-NELFA (Proteintech, 10456-1-AP): https://www.ptglab.com/products/NELF-A-Antibody-10456-1-AP.htm.
anti-NELFC (Cell Signaling Technology, 12265S): https://www.cellsignal.com/products/primary-antibodies/th1l-d5g6w-rabbit-mab/12265.

Eukaryotic cell lines

Policy information about cell lines

Cell line source(s)	Murine mammary tumor cell lines E0771 and E.G7-OVA were purchased from CH3 Biosystems (940001) and ATCC (CRL-2113), respectively. AT-3 and B16-OVA were generated by and generous gifts from Drs. Scott Abrams and Tyler Curiel, respectively. MDA-MB-231 was purchased from ATCC.
Authentication	E0771 and AT-3 cell lines were validated by STR profiling using ATCC cell authentication service (ATCC 137-XVTM). All other cell lines were authenticated by morphology check under microscope.
Mycoplasma contamination	Routine testing for mycoplasma was conducted by MycoAlertTM Mycoplasma Detection Kits (Lonza, 75870-454). All cell lines tested negative for mycoplasma.
Commonly misidentified lines (See ICLAC register)	None.

Animals and other organisms

Policy information about studies involving animals; ARRIVE guidelines recommended for reporting animal research

Laboratory animals	Rag1-/- (stock no. 002216), NSG (stock no. 005557) and C57BL/6CD45.1 congenic mice (stock no. 002014) were purchased from The Jackson Laboratory. Nelfbf/f and distal Lck-cre (dLck-cre) mice were created as previously described [PMID: 19340312, PMID: 22634866]. NelfbTg mice were generated by CRISPR-based gene editing (Cyagen Biosciences). Female mice were used for mammary tumor models, male mice were used for all other tumor models, both female and male mice were used for other ex vivo characterizations. 2.5-10 month old mice were used in all the experiments. Temperature for animal room is set at 72°F, humidity is maintained between 30% - 70%. Animals were maintained under a 12-hour light/12-hour dark cycle.
Wild animals	This study did not involve wild animals.
Field-collected samples	This study did not involve samples collected in the field.
Ethics oversight	All animal protocols were approved by the Institutional Animal Care and Use Committee at The George Washington University.

Note that full information on the approval of the study protocol must also be provided in the manuscript.

ChIP-seq

Data deposition

☒ Confirm that both raw and final processed data have been deposited in a public database such as GEO.

☒ Confirm that you have deposited or provided access to graph files (e.g. BED files) for the called peaks.

Data access links *May remain private before publication.*	To review GEO accession GSE182862: Go to https://www.ncbi.nlm.nih.gov/geo/query/acc.cgi?acc=GSE182862

Files in database submission	scRNA-seq, bulk RNA-seq, Pol II ChIP-seq, ATA-seq of WT/KO CD8+ T cells and NELFB ChIP-seq of WT CD8+ T cells
Genome browser session (e.g. UCSC)	https://www.ncbi.nlm.nih.gov/geo/query/acc.cgi?acc=GSE182862

Methodology

Replicates	Duplicates were used when available.
Sequencing depth	Refer to: https://www.ncbi.nlm.nih.gov/geo/query/acc.cgi?acc=GSE182862
Antibodies	Anti-Pol II (BioLegend; 664906) and anti-NELFB (Cell Signaling Technology;14894S)
Peak calling parameters	Peak calling was done using MACS algorithm.
Data quality	ChIP-seq data quality was checked by MultiQC (v1.9).
Software	MACS, DiffBind, MultiQC, HOMER.

Flow Cytometry

Plots

Confirm that:

- [x] The axis labels state the marker and fluorochrome used (e.g. CD4-FITC).
- [x] The axis scales are clearly visible. Include numbers along axes only for bottom left plot of group (a 'group' is an analysis of identical markers).
- [x] All plots are contour plots with outliers or pseudocolor plots.
- [x] A numerical value for number of cells or percentage (with statistics) is provided.

Methodology

Sample preparation	Detailed protocol for flow cytometry were included in the method section.
Instrument	BD FACSCelesta
Software	FACSDiva and Flowjo
Cell population abundance	No sorting was performed.
Gating strategy	Cells were first gated for FSC/SSC, then doublet exclusion followed by dead cell exclusion. After that, single cells were gated according to the analysis done in specific experimental purpose described in methods or figure legend. Representative gating strategy was shown in extended figure.

- [x] Tick this box to confirm that a figure exemplifying the gating strategy is provided in the Supplementary Information.
