# Supplementary material for: RNA polymerase II pausing factor NELF in CD8+ T cells promotes antitumor immunity
Source: Nat Commun. 2022 Apr 20;13:2155. doi: 10.1038/s41467-022-29869-2 (PMC9021285; doi:10.1038/s41467-022-29869-2)
Supplement: Supplementary file 4 — Description of Additional Supplementary Files [file 41467_2022_29869_MOESM4_ESM.pdf]

Title: Supplementary Data 1

Description: Differentially expressed genes lists (filtered by adjusted *p*-value < 0.05 & FC >= 2) for WT and KO cells at Pre- (Sheet 1) and Post-activation (Sheet 2).
